# Screening of Anti-Obesity Agent from Herbal Mixtures

**DOI:** 10.3390/molecules17043630

**Published:** 2012-03-23

**Authors:** Changhyun Roh, Uhee Jung, Sung-Kee Jo

**Affiliations:** Division of Biotechnology, Advanced Radiation Technology Institute (ARTI), Korea Atomic Energy Research Institute (KAERI), 1266, Sinjeong-dong, Jeongeup, Jeonbuk 580-185, Korea; Email: uhjung@kaeri.re.kr (U.J.); skjo@kaeri.re.kr (S.-K.J.)

**Keywords:** anti-obesity, screening, methyl gallate, HemoHIM, lipid inhibition

## Abstract

Globally, one in three of the World’s adults are overweight and one in 10 is obese. By 2015, World Health Organization (WHO) estimates the number of chubby adults will balloon to 2.3 billion—Equal to the combined populations of China, Europe and the United States. The discovery of bioactive compounds from herbs is one possible way to control obesity and to prevent or reduce the risks of developing various obesity-related diseases. In this study, we screened anti-obesity agents such as methyl gallate from the herbal composition known as HemoHIM that actively inhibits lipid formation as evidenced by Oil Red O staining and triglyceride (TG) contents in 3T3-L1 adipocytes, suggesting their use as an anti-obesity agent. Furthermore, the amount of glycerol released from cells into the medium had increased by treatment of methyl gallate in a concentration-dependent manner. The present study suggests that a promising anti-obesity agent like methyl gallate might be of therapeutic interest for the treatment of obesity.

## 1. Introduction

Obesity is a leading preventable cause of death worldwide, with increasing prevalence in adults and children, and authorities view it as one of the most serious public health challenges of the 21st century. Obesity is one of the most important health problems in human beings and it increases the likelihood of various diseases, such as type 2 diabetes, and particularly heart disease, systemic hypertension, hyperlipidemia, cardiovascular diseases, and certain types of cancer and osteoarthritis [1,2,3,4,5]. The consumption of bioactive compounds from the diet or dietary supplementation is one possible way to control obesity and to prevent or reduce the risks of getting various obesity-related diseases. Recently, there has been a remarkable interest in finding natural lipid inhibitors from natural products to replace synthetic compounds. Natural substances are presumed to be safe since they occur in plant foods, and are therefore seen as more desirable than their synthetic counterparts. The scientific literature shows that natural products contain a large variety of substances that possess lipid inhibition activity. Especially, a variety of herbs from plants have been used as traditional natural medicines for healing many diseases. In particular, various oriental medicinal herbs are reported to have biological activity [6,7,8].

Many scientists have reported that the anti-obesity agents from natural products are a promising field to approach the solution to a global health problem such as obesity [9,10]. A new herbal composition, HemoHIM, was designed to protect the self-renewal tissues and to promote recovery of the immune system [11,12,13] by adding its polysaccharide fraction into a hot water extract of an herb mixture consisting of *Angelica Radix*, *Cnidium Rhizoma* and *Paeoniae Radix* which are listed as edible raw materials in the Korean Food Code. The general composition of HemoHIM was 60.4% carbohydrate, 6% protein and 33.6% other including polyphenols. The bioactive modulating components in HemoHIM were the ethanol-insoluble fraction, and the polysaccharide content in this fraction was 40.9%. In addition, the anti-oxidative components included in the ethanol-soluble fraction of HemoHIM were gallic acid, chlorogenic acid, paeoniflorin, nodakenin and benzoic acid.

Methyl gallate (chemically, the methyl ester of 3,4,5-trihydroxybenzoic acid) belongs to the class of gallic acid-derived compounds. Methyl gallate is a highly potent compound which shows a variety of biological activities. It has been found to be an active ingredient from *Rhus glabra* and *Moutan cortex *where it contributes to their antimicrobial activity. It has also been found to be inhibitory to the growth of intestinal bacteria [14,15]. Furthermore, it shows antioxidant and pro-oxidant properties [16]. We observed that HemoHIM could inhibit fat accumulation in 3T3-L1 cells. It was postulated that at least one compound of HemoHIM was responsible for this lipid inhibition. In this study, we examine the inhibitory effects of methyl gallate from HemoHIM on adipocyte differentiation in 3T3-L1 cells. To the best of our knowledge, this is first report that a bioactive compound such as methyl gallate from HemoHIM has lipid inhibition activity.

## 2. Results and Discussion

**Figure 1:**
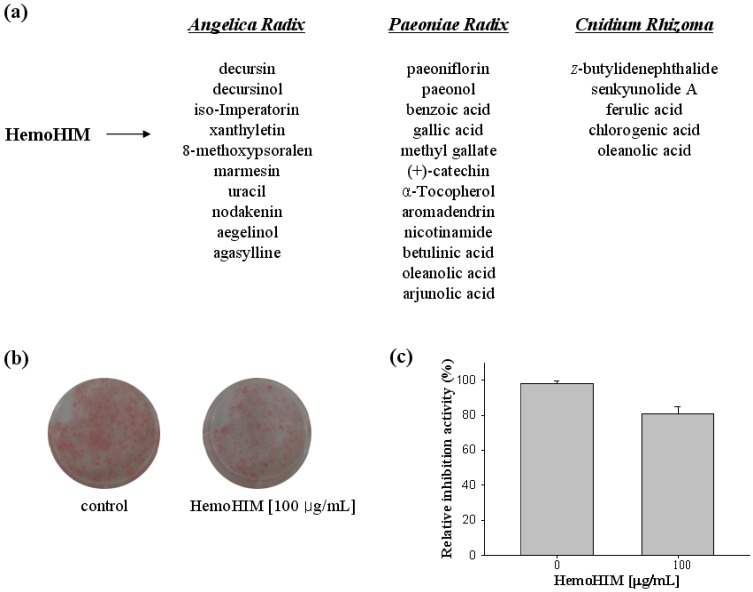
(**a**) Composition of HemoHIM. (**b**) Relative inhibition activity by HemoHIM treatment (**c**) Quantification analysis by Oil Red O staining.

HemoHIM is composed up of the herbal mixtures from *Angelica Radix*, *Cnidium Rhizoma* and *Paeoniae Radix*. In Figure 1a, the main components of the three herbs in HemoHIM are presented. We postulated that at least one compound from three herbs of HemoHIM was responsible for inhibiting lipid accumulation. Among the main compounds examined, methyl gallate showed the highest lipid inhibitory activity. As shown in Figure 1b,c, HemoHIM showed an inhibitory effect at 100 μg/mL concentration on adipocyte differentiation in 3T3-L1 cells. The cell viability by HemoHIM treatment remained at approximately 100% (data not shown).

**Figure 2:**
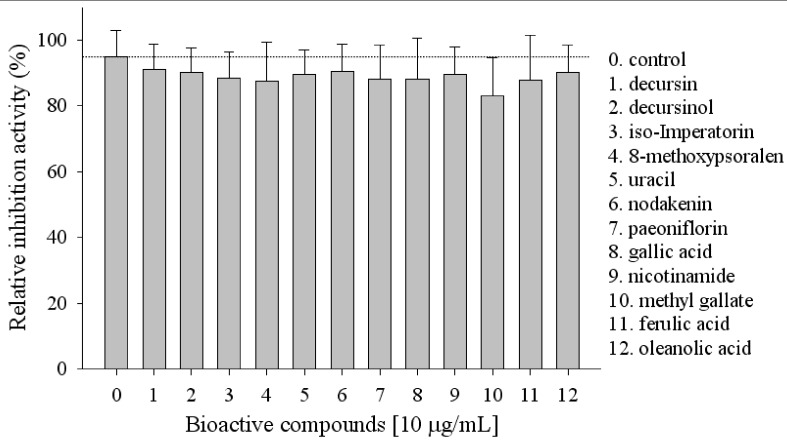
Screening of bioactive compounds with lipid inhibition activity.

The main compounds of the herbal mixtures from *Angelica Radix*, *Cnidium Rhizoma* and *Paeoniae Radix* in HemoHIM were screened. Among the compounds examined decursin, decursinol, iso-imperatorin, 8-methoxypsoralen, uracil, nodakenin, paeoniflorin, gallic acid, nicotinamide, oleanolic acid, ferulic acid and methyl gallate showed high lipid inhibitory activity (Figure 2). 

**Figure 3:**
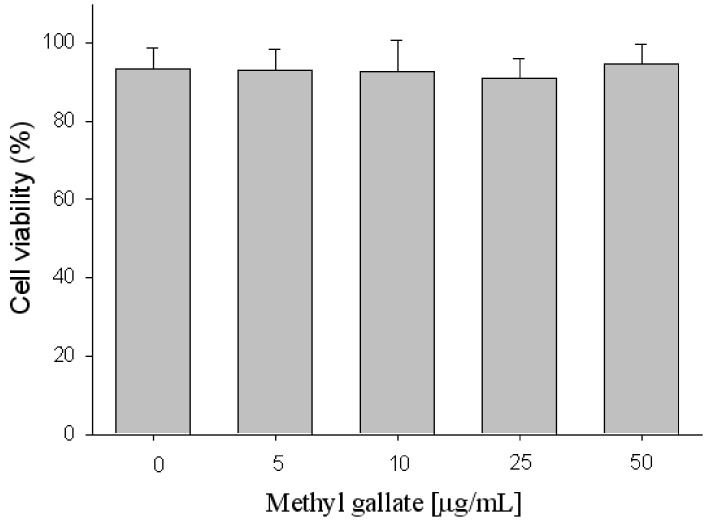
Cell toxicity using the MTT assay.

Methyl gallate showed the highest lipid inhibitory activity. The effects of methyl gallate on cell toxicity in 3T3-L1 preadipocytes were also investigated. Methyl gallate at the doses of 5–50 μg/mL exerted relatively low cytotoxicity on 3T3-L1 preadipocyte cells (Figure 3). During the 2 day incubation period, the viability of preadipocytes and mature adipocytes was shown to be unaffected by 50 μg/mL methyl gallate. In this work, the concentrations of methyl gallate used were therefore from 5 to 50 μg/mL, in the nontoxic range.

We investigated the effect of methyl gallate on the induction of terminal differentiation markers at the end of the differentiation period. Oil Red O staining showed that untreated differentiated cells had many lipid droplets, indicating lipid accumulation, but lipid accumulation was inhibited by methyl gallate treatment in a dose dependent manner. This observation was further supported with the quantitative analysis of neutral lipid content by measuring the absorbance at 500 nm. Untreated and methyl gallate treated differentiated 3T3-L1 cells showed higher levels of lipid staining with the Oil Red O dye, which was significantly reduced by higher doses of methyl gallate treatment.

**Figure 4:**
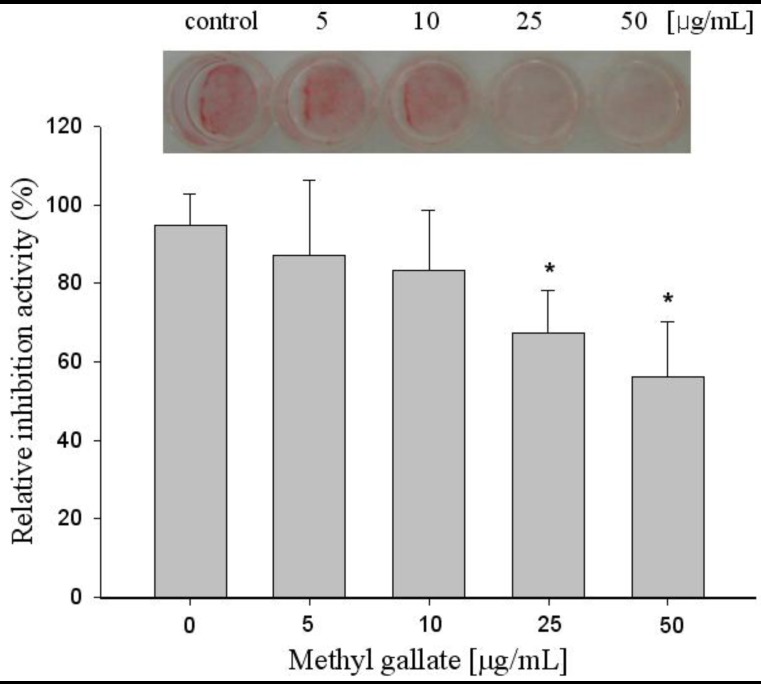
Effects of methyl gallate on lipid inhibition in 3T3-L1 cells. The cells were differentiated with 0, 5, 10, 25, 50 μg/mL of methyl gallate treatment for 8 days and relative inhibition activity by quantification method of Oil Red O staining was examined. Values are expressed as mean ± standard deviation of at least three independent from that of the control treatment. * *p* < 0.01.

The 3T3-L1 adipocytes were cultured and differentiated in DMEM containing 10% FBS for 6 to 8 days under treatment of 5 μg/mL to 50 μg/mL methyl gallate according to differentiating protocols. At 50 μg/mL or less, lipid accumulation in differentiated adipocytes was dose-dependently attenuated, as evidenced by Oil Red O staining. Effects of methyl gallate on the intracellular lipid accumulation in 3T3-L1 adipocytes are shown in Figure 4. Incubation of 3T3-L1 cells with 5, 10, 25 and 50 μg/mL methyl gallate decreased the lipid droplets by 12, 17, 33, and 44%, respectively. This suggests that the treatment with methyl gallate can reduce adipogenesis significantly in 3T3-L1 cells. Intracellular lipid accumulation was quantified to adjusted cell numbers at the end of the treatment and methyl gallate not only impaired lipid droplet formation during differentiation, but also reduced lipid content in adipocytes significantly within 2 days by about 44% compared to fully differentiated 3T3-L1 cells.

**Figure 5:**
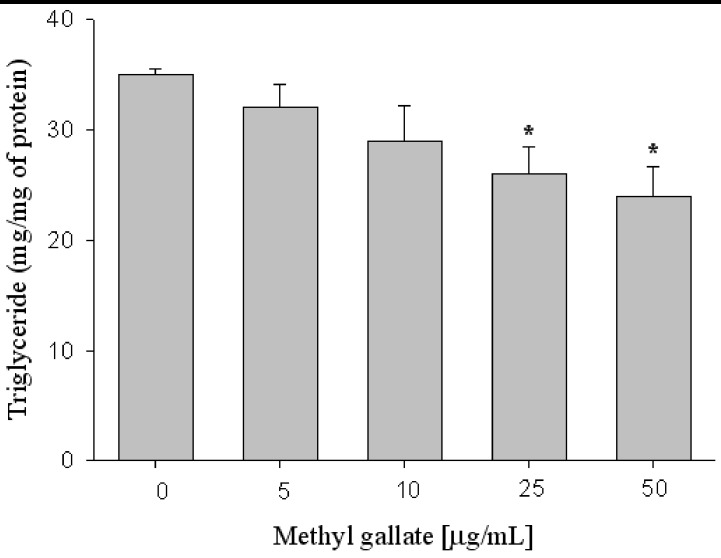
Effects of methyl gallate on adipocyte differentiation in 3T3-L1 cells. Cells were cultured in the medium containing methyl gallate, and lipid accumulation was measured by triglyceride (TG) content. X: concentration Y: Triglyceride (mg/mg protein). Values are expressed as mean ± standard deviation of at least three independent from that of the control treatment. * *p* < 0.01.

As shown in Figure 5, lipid accumulation was measured based on the triglyceride (TG) contents of 3T3-L1 cells differentiated with methyl gallate treatment. The amount of TG in 3T3-L1 cells was decreased approximately up to 26 and 33% in the presence of 25 and 50 μg/mL methyl gallate (Figure 5). 

**Figure 6:**
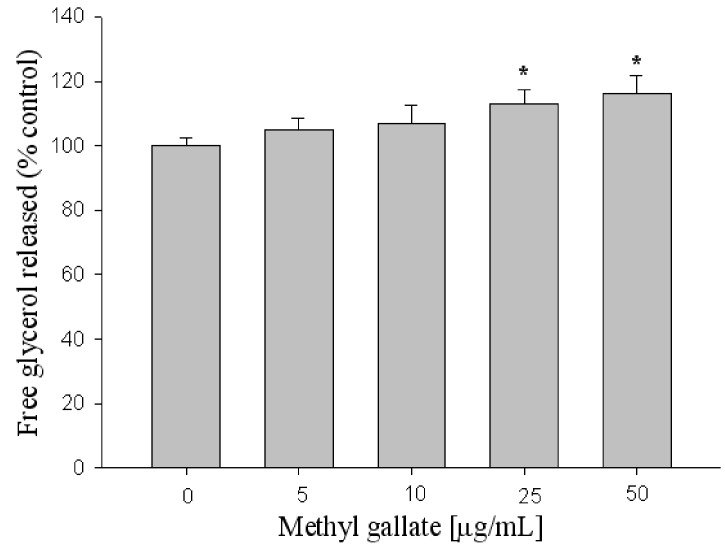
Effect of methyl gallate screened on lipolysis in 3T3-L1 adipocytes. X: methyl gallate, Y: Glycerol content (mM). Values are expressed as mean ± standard deviation of at least three independent from that of the control treatment. * *p* < 0.01.

To estimate the lipolysis effect of methyl gallate, the amount of glycerol released into the medium was measured. The amount of glycerol in the medium was increased by 20% in the presence of 50 μg/mL methyl gallate (Figure 6). In the present study, it was clear that treatment with methyl gallate decreased the intercellular lipid content in 3T3-L1 adipocytes, and increased the amount of glycerol released into the medium, indicating activation of lipolysis.

Methyl gallate from HemoHIM thus showed the highest lipid inhibition activity suggesting potential anti-obesity activity and might be of therapeutic interest with respect to the treatment of obesity.

## 3. Experimental

### 3.1. Materials

The dried and powdered HemoHIM was extracted three times with distilled water and ethanol, and extracts were obtained by removal of the solvent in evaporation. The extracts were stored at −20 °C for further study. Methyl gallate was purchased from Sigma-Aldrich Chemical Co. (St. Louis, MO, USA). All reagents were of the highest grade available. 

### 3.2. Screening of Bioactive Compound with Anti-obesity Activity and Cell Culture and Differentiation

The bioactive compounds from *Angelica Radix*, *Cnidium Rhizoma* and *Paeoniae Radix* were screened as anti-obesity agents. 3T3-L1 preadipocytes were obtained from the American Type Culture Collection (ATCC) (Manassas, VA, USA). 3T3-L1 preadipocytes were grown in Dulbecco’s Modified Eagle Medium (DMEM) supplemented with 10% (v/v) heat-inactivated Fetal Bovine Serum (FBS) at 37 °C in an atmosphere containing 5% CO_2_. To induce adipocyte differentiation, 2-day post-confluent 3T3-L1 preadipocytes were stimulated for 48 h with MDI inducer (0.5 mM 3-isobutyl-1-methylxanthine, 2.5 μM dexmethansone and 10 μg/mL insulin,) including HemoHIM or methyl gallate, and then maintained in DMEM supplemented with 10% FBS and 10 μg/mL insulin including HemoHIM or methyl gallate for 6 days. 3T3-L1 cells were treated with HemoHIM or methyl gallate in DMEM supplemented with 10% FBS for 2 days. To examine the effect of HemoHIM or methyl gallate on adipocyte differentiation, the medium and HemoHIM or methyl gallate were treated every 2 days until the end of the experiment on day 8. 

### 3.3. Cell Viability and Oil Red O Staining

Cell toxicity was determined colorimetrically using the MTT assay [17]. Cells cultured in DMEM medium were treated with methyl gallate (0, 5, 10, 25, and 500 μg/mL) for 2 days and then treated by 5 mg/mL MTT (3-(4,5-dimethyl-2-thiazolyl)-2,5-diphenyltetrazolium bromide) solution (Sigma) for 3 h. After cells were dissolved in 0.04 N HCl (in isopropanol), formazan level was analyzed by measuring OD 570 nm (against OD 630 nm). Oil Red O staining was performed as previously described [18]. 3T3-L1 adipocytes were washed with Phosphate-Buffered Saline (PBS) and fixed with 10% formalin for 30 min. After two washes with distilled water, cells were stained for at least 1 h at room temperature in freshly diluted Oil Red O containing 0.5% Oil Red O in isopropanol. Finally, the dye retained in the 3T3-L1 cells was eluted with isopropanol and quantified by measuring the optical absorbance at 500 nm.

### 3.4. Measurement of Triglyceride (TG) and Glycerol Released

Cellular triglyceride contents were measured using a commercial TG assay kit (Asan Pharm. Co., Seoul, Korea) according to the manufacturer’s instructions. Cells were treated with methyl gallate with concentrations of 0, 5, 10, 25, and 50 μg/mL in 6 well plates during adipocyte differentiation for 6 days. The cells were washed twice with PBS, scraped in 75 μL of homogenizing solution (154 mM KCl, 1 mM EDTA and 50 mM Tris, pH 7.4) and sonicated to homogenize the cell suspension. The residual cell lysate was centrifuged at 3000 rpm for 5 min at 4 °C to remove fat layers. The supernatants were assayed for TG and protein contents. TG was normalized to protein concentration determined by the Bovine Serum Albumin (BSA) as standard. Results were expressed as milligrams of TG per milligram of cellular protein. Lipolysis was assessed by the measurement of glycerol released (Free Glycerol Reagent, Sigma) into the medium, according to the manufacturer’s instruction. Effects of methyl gallate on glycerol release in 3T3-L1 adipocytes were examined. The differentiated 3T3-L1 adipocytes were treated in serum-free medium with methyl gallate (0, 5, 10, 25, and 50 μg/mL) for 24 h. The medium was collected and assayed for glycerol content. Values are expressed as mean ± standard error of at least three independent experiments, each performed in triplicate (n = 3). 

### 3.5. Statistics Data Analysis

Statistical analysis was done by using one way analysis of variance (ANOVA). *p* value (* *p* < 0.01) was considered as significant.

## 4. Conclusions

In summary, we screened an anti-obesity agent from HemoHIM which has promising activity. It would be interesting to examine the main compound which has shown higher activity than HemoHIM. The present study suggests that methyl gallate might be of therapeutic interest with respect to the treatment of obesity. 

## 5. Implications

The discovery of bioactive compounds from diet or dietary supplementation is one of possible ways to control obesity and to prevent or reduce the risks of various obesity-related diseases. These research results support the notion that methyl gallate from HemoHIM potently inhibits lipid formation, suggesting its use as an anti-obesity agent.
